# Relationship between social activities and cognitive impairment in Chinese older adults: the mediating effect of depressive symptoms

**DOI:** 10.3389/fpubh.2024.1506484

**Published:** 2025-01-24

**Authors:** Qianke Yang, Shichong Lin, Zhuyun Zhang, Shuhao Du, Dan Zhou

**Affiliations:** ^1^Department of Physical Medicine and Rehabilitation, The Second Affiliated Hospital and Yuying Children's Hospital of Wenzhou Medical University, Zhejiang, China; ^2^School of Smart Health Care (School of Health & Medical), Zhejiang Dongfang Polytechnic, Zhejiang, China; ^3^Department of Critical Care Medicine, Shenzhen People’s Hospital, First Affiliated Hospital of Southern University of Science and Technology, The Second Affiliated Hospital of Jinan University, Shenzhen, China

**Keywords:** social participation, depressive symptoms, cognitive ability, aging demographic, CHARLS

## Abstract

**Background:**

The differential impacts of various social activities on dementia prevention and the mediating role of depression in this relationship remain unclear.

**Objectives:**

This study aimed to identify the effects of different social activities on cognitive function, examine the mediating role of depression, and provide evidence for targeted interventions to prevent cognitive decline.

**Methods:**

Using data from CHARLS, we employed RCS analysis and Structural Equation Modeling to examine the relationships between social activities, depression, and cognitive function in older adults.

**Results:**

Social activity participation is non-linearly inversely related to both cognitive impairment and depression risk. Cognitive function and social activities were significantly mediated by depression. Cognitively stimulating and physically stimulating activities had the greatest positive effects on mental health.

**Conclusion:**

Our findings highlight the complex interplay between social engagement, depression, and cognitive health in aging. They support developing targeted interventions promoting physical and cognitive social activities to maintain cognitive function and reduce depression risk in older adults, potentially alleviating the burden of cognitive impairment in aging populations.

## 1 Introduction

Dementia and cognitive decline pose serious health risks to the world’s aging population, placing a heavy strain on patients, families, and healthcare systems (1, 2). As the global population ages, these issues are becoming increasingly prominent (3). China is the world’s most populous country with a rapidly aging demographic, with projections indicating this number could be over 10 million by 2060 (4).

While effective treatments for cognitive disorders and dementia remain elusive, growing evidence suggests that social engagement may play a crucial role in early identification and prevention of cognitive decline (5, 6). However, existing research presents conflicting findings. Several studies have demonstrated positive correlations between leisure-time social interactions and cognitive function in older adults (7, 8), while others have failed to establish significant associations (9, 10). While previous studies have examined the connection between social participation, depression, and the CHARLS data (11, 12), a critical knowledge gap exists regarding the ways in which the frequency of participation in different social activities affects mental health and cognitive abilities in different ways. This study addresses this significant gap by examining the effects of participation frequency across diverse social activities, providing a more nuanced understanding of the complex relationship between social engagement, depression, and cognitive function in older adults.

Moreover, depression, a common mental health concern among aging adults (13), may serve as a mediating factor in the relationship between social engagement and cognitive function (12). As social participation gains prominence in depression research, it becomes imperative to deepen our understanding of how older adults adapt to declining physical function and changing social roles, how this adaptation process influences their mental health, and the subsequent impact of mental health issues, particularly depression, on cognitive abilities in the older adult. The complex interplay between social engagement, depression, and cognitive function in older adults remains inadequately explored, particularly in the context of diverse social activities. This gap in knowledge hinders the development of targeted interventions for maintaining cognitive health in aging populations.

In order to fill in these important knowledge gaps, the current study will use longitudinal data from the nationally representative CHARLS survey to assure the validity and generalizability of findings. It will also examine the relationships between various social activity types and the trajectories of cognitive function in older Chinese adults, as well as the mediating role of depression in this relationship.

This study predict that higher levels of social activity participation will be linked to lower depression scores and higher Mini-Mental State Examination (MMSE) (14) scores based on the body of research and theoretical frameworks. Additionally, it’s anticipated that depression (measured by the Epidemiologic Studies Depression Scale, CES-D 10) (15) will somewhat mitigate the link between engagement in social activities and cognitive performance.

By elucidating these relationships, this study seeks to provide empirical evidence for developing targeted interventions to prevent cognitive decline. These findings can inform strategies aimed at promoting healthy aging, as well as reducing societal burdens associated with cognitive impairment and dementia among older adults. This research is particularly timely and relevant given the rapidly aging population in China and the increasing global focus on maintaining cognitive health in later life.

## 2 Method

### 2.1 Data source and study population

This study utilized data from CHARLS (11) waves conducted in 2011, 2013, 2015, and 2018. CHARLS is a longitudinal survey that is nationally representative and gathers extensive data on the lifestyle, health, and socioeconomic situation of middle-aged and older Chinese citizens. Its comprehensive design and longitudinal nature, combined with its rich dataset, make it particularly suitable for studying aging-related changes and the complex interplay between social, health, and economic factors. Although 2020 is the latest wave of CHARLS data, the COVID-19 pandemic led to significant data missingness in this wave. Therefore, we chose to use the previous four waves for this study. The Peking University Biomedical Ethics Review Committee authorized the study protocol (IRB00001052-11015), and all subjects gave their informed permission.

#### 2.1.1 Participants were included if they met the following criteria

Participants included in this study were aged 45 years or older and had completed both the MMSE and the CES-D 10 (15) assessments in 2018. The cutoff age of 45 years was chosen to align with the study’s focus on middle-aged and older adults, ensuring the inclusion of individuals at risk of early cognitive decline and depression while also capturing a wide range of social engagement patterns relevant to the aging process. Additionally, they had participated in at least one social activity across the four survey waves and provided complete data on key variables. These criteria align with prior research guidelines on aging and mental health assessments.

**Figure 1:**
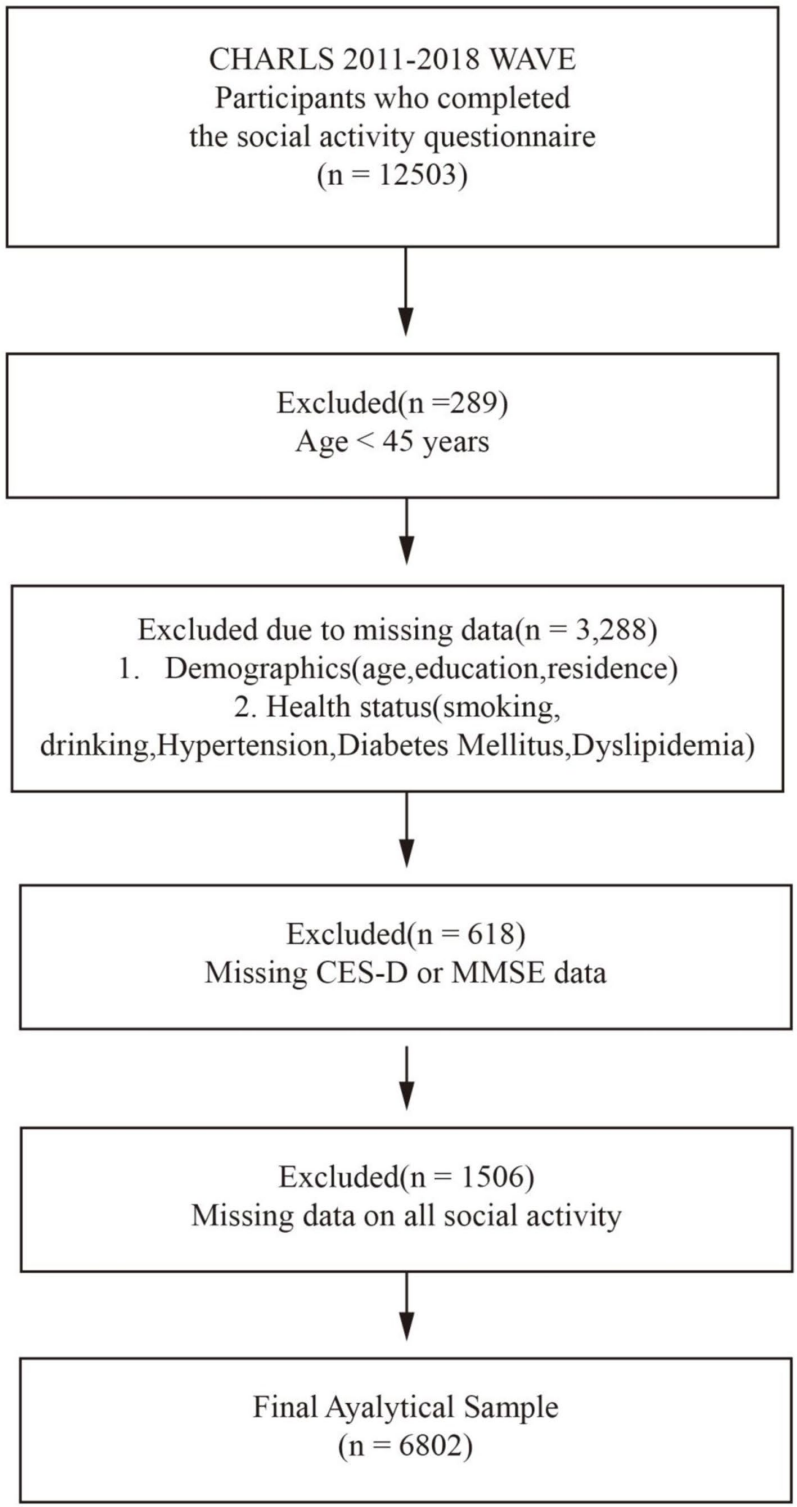
Flowchart of analytic sample.

From an initial sample of 12,503 individuals, 6,802 met the inclusion criteria (Figure 1). Exclusions were due to: age < 45 years (*n* = 289), missing data on key demographic and health variables (*n* = 3,288), incomplete MMSE or CES-D 10 in 2018 (*n* = 618), and missing all data on social activity participation across all four waves (*n* = 1,506).

### 2.2 Measures

#### 2.2.1 Independent variable: social activity participation

Social activity participation was assessed through nine specific activities, including having conversations with friends (Social 1), participating in community clubs (Social 2) or games such as chess, cards, or mahjong (Social 3), assisting loved ones or acquaintances who did not reside with the participant (Social 4), visiting social or athletic clubs (Social 5), participating in community-related organizations (Social 6), performing volunteer or altruistic work, caring for ill or disabled adults outside the household (Social 7), engaging in training or instructional programs (Social 8), and investment in stocks (Social 9). Participation frequency for each activity was rated on a four-point scale: never (0), not regularly (1), almost every week (2), or almost daily (3) (16–18). For each activity, we calculated a total score across the four survey waves and summed these to derive an overall score, ranging from 0 to 108. Higher scores indicated greater levels of social engagement.

#### 2.2.2 Mediating variable: depression

Depression was assessed using the CES-D 10. Scores range from 0 to 30, with higher scores indicating more severe depressive symptoms. A cut-off score of >10 was used to categorize participants into depressed and non-depressed groups (19).

#### 2.2.3 Dependent variable: cognitive function

Using MMSE assesses orientation, attention, calculation, recall, and language abilities. Participants were classified into cognitive impairment and non-impairment groups based on education-adjusted cut-off points.

#### 2.2.4 Potential covariates

To account for potential confounding factors, we included the following control variables in the analysis: age, sex, education level (no formal education, primary education, middle school, college) (20), and residence (rural or urban) (21), as they are fundamental demographic factors that could influence social activity participation and health outcomes. Lifestyle factors, including smoking status and alcohol consumption, were included because they are known to impact physical and mental health and may confound the relationship between social activity participation and the dependent variables. Additionally, chronic conditions such as hypertension, diabetes, and dyslipidemia (coded as binary variables: diagnosed or undiagnosed) were controlled for because these conditions can affect overall health and potentially interact with both social activity and the outcomes of interest (22–24). By including these variables, we aimed to minimize bias and better isolate the association between social activity participation and the outcomes of interest.

#### 2.2.5 Statistical analysis

R software was used for all studies, along with the dedicated ‘charlsr’ package for processing CHARLS data. Using methods from the ‘charlsr’ package, data preparation involves extracting and combining pertinent variables from the 2011, 2013, 2015, and 2018 CHARLS waves.

Calculations of means, standard deviations, frequencies, and percentages were made for the following descriptive statistics: depressive status, cognitive function, social activity involvement, and demographic data.

Using a number of sophisticated statistical tools, we investigated the connections between depression, cognitive performance, and social activity involvement. RCS analysis was utilized to investigate potential non-linear relationships between these variables.

To account for potential heterogeneity in the population, we conducted stratified analyses based on key demographic characteristics such as age, sex, and education level. These subgroup analyses explored potential differences in the effect of social activity on cognitive function across various population segments. Forest plots were used to display effect sizes and confidence intervals for different subgroups, facilitating easy comparison of results across strata.

Finally, we conducted detailed analyses of nine specific types of social activities as well as total social activity frequency scores. We analyzed each activity type and total social activity frequency score separately using Structural equation modeling (SEM) (25) to explore their relationship with depression and cognitive functioning.

## 3 Result

### 3.1 Baseline characteristics

The 7,019 participants had a mean age of 57.71 ± 8.39 years, with 55.27% of them being female. The baseline covariate distribution, dependent variable distribution, and mediating variable distribution (Table 1).

**Table 1:** Sample characteristics of participants at baseline.

Variable	Total
Independent variable	
social01	3.54 ± 2.90
social02	1.65 ± 2.70
social03	0.68 ± 1.13
social04	0.76 ± 1.86
social05	0.12 ± 0.57
social06	0.06 ± 0.35
social07	0.14 ± 0.58
social08	0.03 ± 0.23
social09	0.04 ± 0.55
social_total	7.01 ± 5.39
Mediating variable	
CES-D 10	
Depression	2,611(38.39)
Normal	4,191(61.61)
CES-D score	8.74 ± 6.48
Dependent variable	
Cognitive impairment	
No	1766(25.96)
Yes	5,036(74.04)
MMSE	14.86 ± 6.47
Control variables	
Age	57.71 ± 8.39
Sex	
Female	3,758(55.27)
Male	3,041(44.73)
Education	
College or above	117(1.72)
Middle school	2,201(32.36)
Primary school	1,555(22.86)
No formal education	2,929(43.06)
Residence	
Rural	4,389(64.53)
Urban	2,413(35.47)
Smoke	
No	4,764(70.04)
Yes	2038(29.96)
Drink	
No	4,538(66.72)
Yes	2,264(33.28)
Hypertension	
No	4,198(61.72)
Yes	2,604(38.28)
DM_test	
No	5,981(87.93)
Yes	821(12.07)
Dyslipidemia	
No	3,961(58.23)
Yes	2,841(41.77)

DM_test, diabetes.

### 3.2 Sensitivity analysis

The sensitivity analysis revealed significant differences between participants included in the study (*n* = 6,802) and those excluded (*n* = 5,701), providing crucial insights into the generalizability of our findings (Table 2). The included group demonstrated significantly higher participation rates across most social activity types, particularly in activities social01 through social06. This may indicate that the older population in our study sample is more socially active than the general population, which might cause the influence of social activities to be overvalued.

**Table 2:** Sensitivity analysis.

Variable	Total (*n* = 12,503)	Include (*n* = 6,802)	Exclude (*n* = 5,701)	Statistic	*p*-value
Age	57.93 ± 9.16	57.71 ± 8.39	58.19 ± 10.01	2.84	**<0.01**
Sex				1.86	0.17
Female	6,836(54.71)	3,758(55.27)	3,078(54.04)		
Male	5,659(45.29)	3,041(44.73)	2,618(45.96)		
Education				3.42	0.33
College or above	235(1.88)	117(1.72)	118(2.07)		
Middle school	3,847(30.78)	2,201(32.36)	1,646(28.90)		
No formal education	5,627(45.03)	2,929(43.06)	2,698(47.37)		
Primary school	2,788(22.31)	1,555(22.86)	1,233(21.65)		
Residence				5.33	**0.02**
Rural	7,953(63.61)	4,389(64.53)	3,564(62.52)		
Urban	4,550(36.39)	2,413(35.47)	2,137(37.48)		
Smoke				0.34	0.56
No	8,785(70.26)	4,764(70.04)	4,021(70.53)		
Yes	3,718(29.74)	2038(29.96)	1,680(29.47)		
Drink				3.98	0.05
No	8,438(67.49)	4,538(66.72)	3,900(68.41)		
Yes	4,065(32.51)	2,264(33.28)	1801(31.59)		
Hypertension				14.47	**<0.001**
No	7,902(63.23)	4,198(61.72)	3,704(65.03)		
Yes	4,596(36.77)	2,604(38.28)	1992(34.97)		
DM_test				1.05	0.31
No	8,064(88.14)	5,981(87.93)	2083(88.75)		
Yes	1,085(11.86)	821(12.07)	264(11.25)		
Dyslipidemia				647.74	**<0.0001**
No	8,452(67.94)	3,961(58.23)	4,491(79.64)		
Yes	3,989(32.06)	2,841(41.77)	1,148(20.36)		
social01	2.77 ± 2.88	3.54 ± 2.90	1.86 ± 2.58	−34.13	**<0.0001**
social02	1.28 ± 2.44	1.65 ± 2.70	0.84 ± 1.99	−19.29	**<0.0001**
social03	0.54 ± 1.05	0.68 ± 1.13	0.36 ± 0.91	−17.46	**<0.0001**
social04	0.63 ± 1.72	0.76 ± 1.86	0.48 ± 1.53	−9.17	**<0.0001**
social05	0.10 ± 0.53	0.12 ± 0.57	0.08 ± 0.49	−4.54	**<0.0001**
social06	0.05 ± 0.32	0.06 ± 0.35	0.03 ± 0.28	−4.53	**<0.0001**
social07	0.11 ± 0.52	0.14 ± 0.58	0.08 ± 0.45	−6.4	**<0.0001**
social08	0.02 ± 0.24	0.03 ± 0.23	0.02 ± 0.24	−0.75	0.46
social09	0.04 ± 0.52	0.04 ± 0.55	0.04 ± 0.49	−0.21	0.84
social_total	5.55 ± 5.52	7.01 ± 5.39	3.80 ± 5.16	−34.02	**<0.0001**
MMSE	14.50 ± 6.48	14.86 ± 6.47	13.99 ± 6.47	−7.11	**<0.0001**
Cognitive impairment				37.02	**<0.0001**
No	2,783(23.93)	1,766(25.96)	1,017(21.06)		
Yes	8,848(76.07)	5,036(74.04)	3,812(78.94)		
CES-D 10	8.80 ± 6.56	8.74 ± 6.48	8.88 ± 6.67	1.05	0.29
CES-D score				37.02	**<0.0001**
Depression	4,497(38.94)	2,611(38.39)	1,886(39.73)		
Normal	7,052(61.06)	4,191(61.61)	2,861(60.27)		

DM_test, diabetes. Bold values indicate statistical significance (*p* < 0.05).

Demographically, the included group was slightly younger (57.71 ± 8.39 vs. 57.93 ± 9.16 years), and had a slightly higher proportion of females (55.27% vs. 44.73%). Interestingly, education levels did not differ significantly between the groups (*p* = 0.33). These disparities indicate that our findings may be more applicable to slightly older and more female older adult populations. Notably, the included group had significantly higher rates of diabetes, possibly reflecting a greater tendency among those with health issues to participate in surveys or social activities, or a heightened awareness of their health status.

These differences unveil potential selection bias, indicating that study participants may be healthier, more active, and cognitively better functioning. This “healthy participant effect” might lead to an overestimation of the protective effect of social activities on cognitive function. Furthermore, the significant differences between included and excluded groups may limit the generalizability of our results, potentially making them more applicable to healthier, more socially engaged older adult populations.

These findings resonate with our main analysis results. For instance, the positive correlation we found between total social activity scores and cognitive function is consistent with the higher social engagement and better cognitive function observed in the included group. Indeed, the included group showed slightly higher MMSE scores (14.86 ± 6.47 vs. 13.99 ± 6.47, *p* < 0.0001).

It’s worth noting that the included group showed slightly lower depression scores as measured by CES-D 10 (8.74 ± 6.48 vs. 8.88 ± 6.67, *p* = 0.29), although this difference is not statistically significant and relatively small.

Given these limitations, caution is warranted in interpreting our results. While our study provides valuable insights into the relationships between social activities, depression, and cognitive function, these findings may primarily apply to older adult populations with higher social engagement and better health status. Future research should focus on older adult groups with lower social participation and poorer health conditions to gain a more comprehensive understanding.

Methodologically, these findings offer important implications. In our main analysis, we need to adequately control for potential confounding factors such as baseline cognitive function and chronic health conditions. Additionally, considering weighted analyses or propensity score matching might help reduce the impact of selection bias and enhance the reliability and generalizability of our results.

In conclusion, this sensitivity analysis not only reveals important sample differences but also provides valuable context for understanding and interpreting our main research findings. It underscores the complexity of conducting large-scale longitudinal studies in older adult populations and highlights the numerous factors that need to be considered when interpreting and generalizing research results in this field.

The comprehensive analysis of social activity frequency and cognitive function across diverse subgroups of older Chinese adults consistently revealed protective effects (Table 3), reinforcing our study’s main findings. Higher social activity participation was associated with lower cognitive impairment risk across various demographic and health-related categories, including education levels, genders, residence types, and chronic condition statuses.

**Table 3:** Association between total social score and risk of all-cause dementia in middle-aged and older adults.

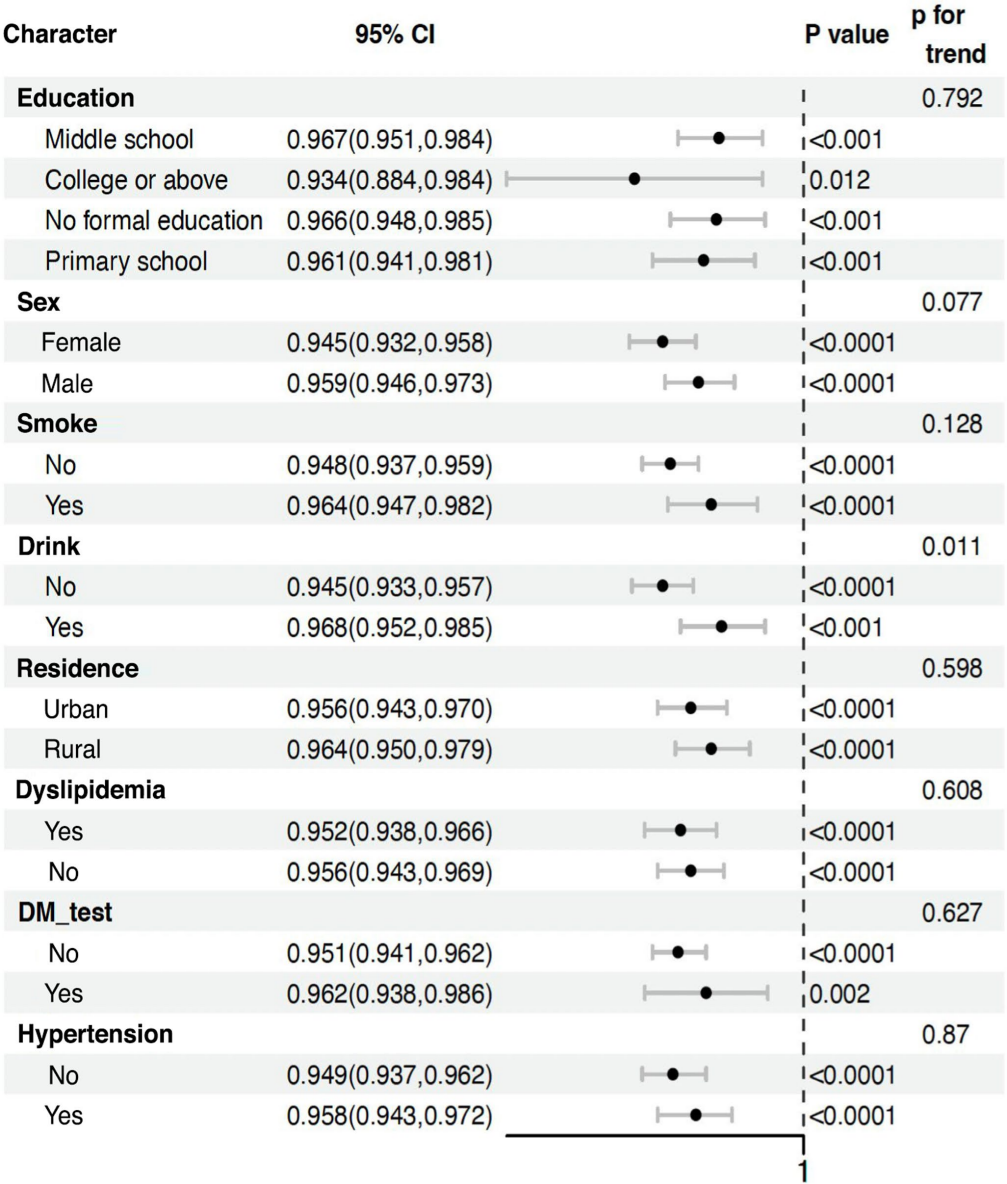

DM_test, diabetes.

Notably, the protective effects were observed across all subgroups, with only slight variations. Among education levels, all categories showed similar protective effects, with odds ratios ranging from 0.933 to 0.967. Males (OR: 0.960, 95% CI: 0.947–0.974) showed a marginally stronger protective effect compared to females (OR: 0.945, 95% CI: 0.932–0.958). Interestingly, smokers (OR: 0.967, 95% CI: 0.949–0.984) and drinkers (OR: 0.970, 95% CI: 0.954–0.987) demonstrated slightly stronger protective effects compared to non-smokers and non-drinkers, respectively. Rural residents (OR: 0.967, 95% CI: 0.953–0.981) exhibited a marginally stronger effect compared to urban residents (OR: 0.955, 95% CI: 0.942–0.968).

The persistence of these effects, regardless of chronic condition status, highlights the potential of social activities as a universal strategy for cognitive health maintenance in the older adult. This is evidenced by the similar odds ratios for those with and without hypertension, diabetes (DM_test), and dyslipidemia.

These findings align with our primary analysis, demonstrating a significant inverse relationship between social activity participation and cognitive decline risk. They provide a nuanced understanding of how this relationship manifests across different population segments, suggesting the robustness of social activity benefits across various demographic and health profiles.

Importantly, the *p* for trend values indicate that the protective effect of social activities does not significantly differ across most subgroups (education: *p* = 0.821, residence: *p* = 0.363, dyslipidemia: *p* = 0.405, DM_test: *p* = 0.877, hypertension: *p* = 0.99). However, there’s a marginally significant trend for gender (*p* = 0.053) and a significant trend for drinking status (*p* = 0.004), suggesting that the effect of social activities might vary more substantially across these categories. While these results offer robust support for our conclusions, it’s important to consider limitations such as varying sample sizes across subgroups. Nevertheless, in the context of China’s rapidly aging population, these findings have significant implications for public health strategies, suggesting that promoting social activities could be a widely applicable approach to maintaining cognitive health in diverse older adult populations. The consistency of protective effects across various subgroups underscores the potential of social engagement as a universal intervention for cognitive health preservation in older adults.

### 3.3 Relationship between social activity and cognitive impairment risk

**Figure 2:**
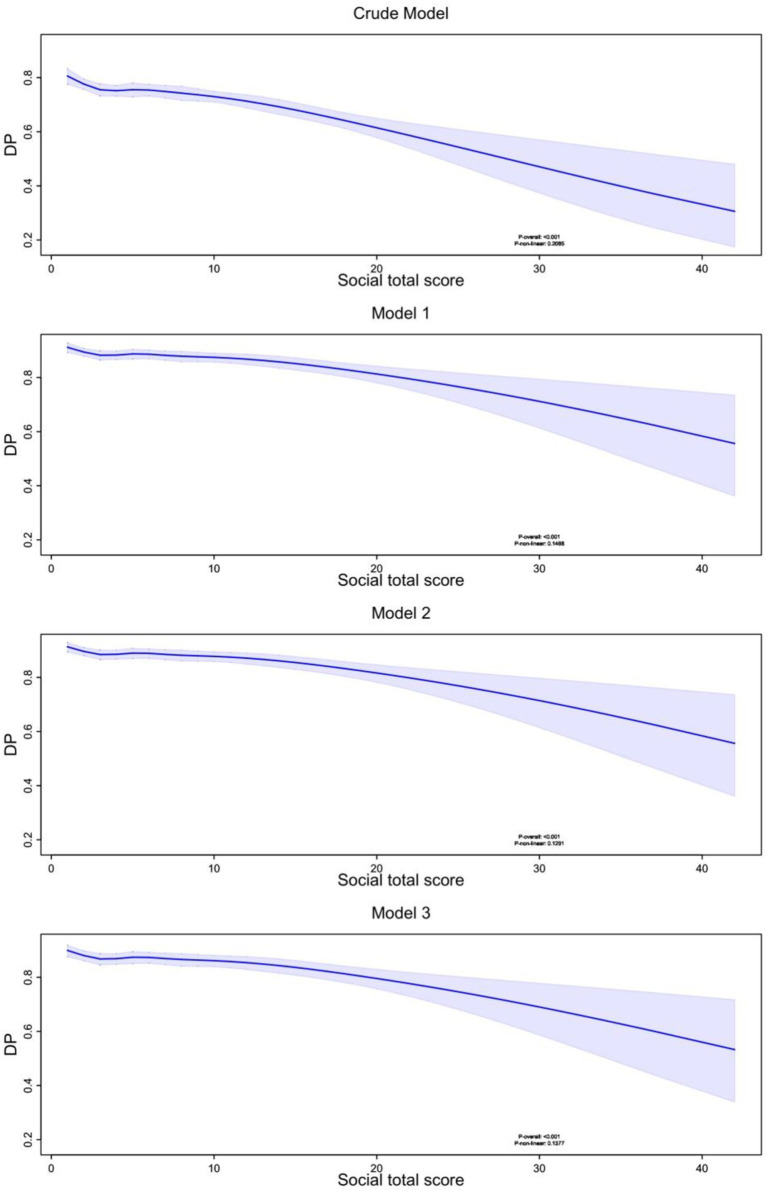
Nonlinear relationship between social total activity score and dementia probability. RCS modeling with 6 knots was used to examine the nonlinear relationship between total social activity scores and DP across four models: a crude model and three progressively adjusted models accounting for different sets of confounders. In all models, a negative association between total social activity scores and dementia probability was observed, with higher activity scores corresponding to lower dementia probability. The shaded areas represent 95% confidence intervals, and the *p*-values indicate the significance of the nonlinear relationship. DP, Dementia Probability.

A significant negative linear association between social activity and cognitive impairment risk was consistently demonstrated by the results (Figure 2). This relationship remained robust across all models (P-overall <0.001), with cognitive impairment probability decreasing from approximately 0.8 to 0.4 as social activity scores increased from 0 to 40. The relationship showed no significant non-linear pattern (P-non-linear >0.05 in all models, ranging from 0.1291 to 0.2085), indicating a steady decline in cognitive impairment risk with increasing social activity participation.

These findings suggest that increasing social activity participation could be an effective strategy for cognitive impairment prevention, with consistent benefits across different levels of social engagement. The widening confidence intervals at higher activity scores (>30) indicate a need for cautious interpretation of effects at these extremes, possibly due to fewer participants with very high social activity levels.

### 3.4 Relationship between total social activity score and depression score

**Figure 3:**
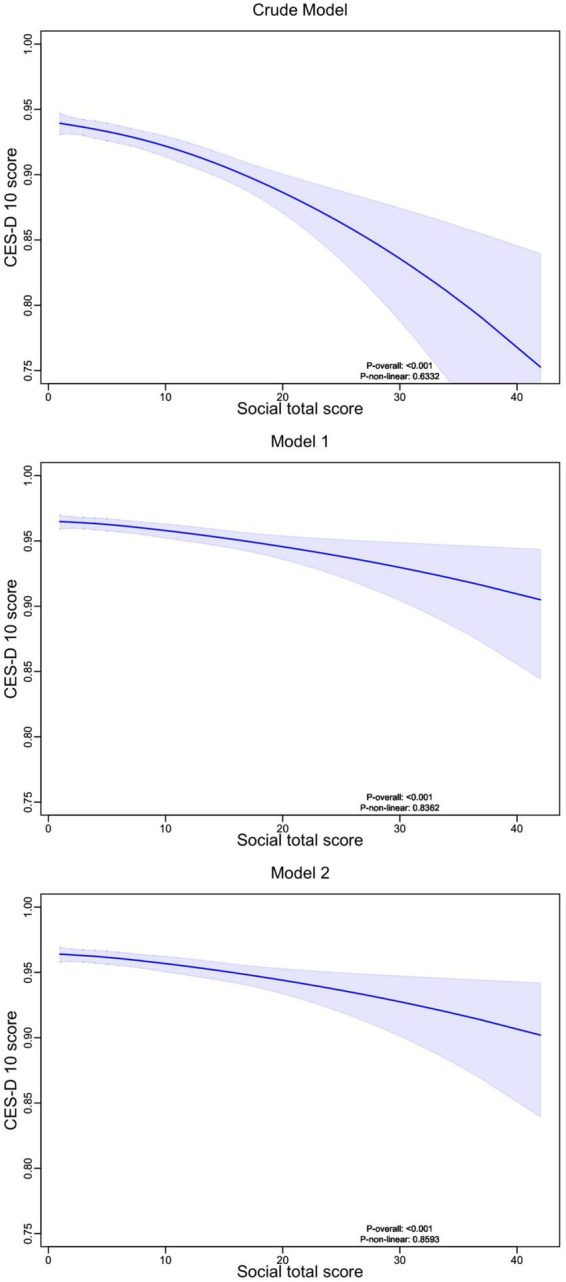
Nonlinear relationship between social total activity score and depression scores. RCS analysis with 4 knots was used to explore the nonlinear relationship between total social activity scores and CES-D 10 score. The analysis included three models: an unadjusted Crude Model, Model 1 adjusted for demographic characteristics, and Model 2 further adjusted for lifestyle factors and chronic diseases. Across all models, higher social total scores were associated with lower depression scores, with shaded regions representing the 95% confidence intervals. The *p*-values indicate the significance of the nonlinear trend.

According to the results (Figure 3), depression scores showed a positive association with cognitive impairment risk across all models, indicating a significant link between increased depression and higher cognitive impairment probability. This relationship was significant across all models (P-overall <0.001).

The graphs displayed non-linear features (P-non-linear <0.001), with a steeper increase in cognitive impairment risk at lower depression scores (0–10 points) and a more gradual increase at higher scores (10–30 points). As depression scores increased from 0 to 30, the probability of cognitive impairment increased from approximately 0.60 to 0.90, with the relationship remaining consistent across all adjusted models.

### 3.5 Relationship between depression and cognitive impairment

**Figure 4:**
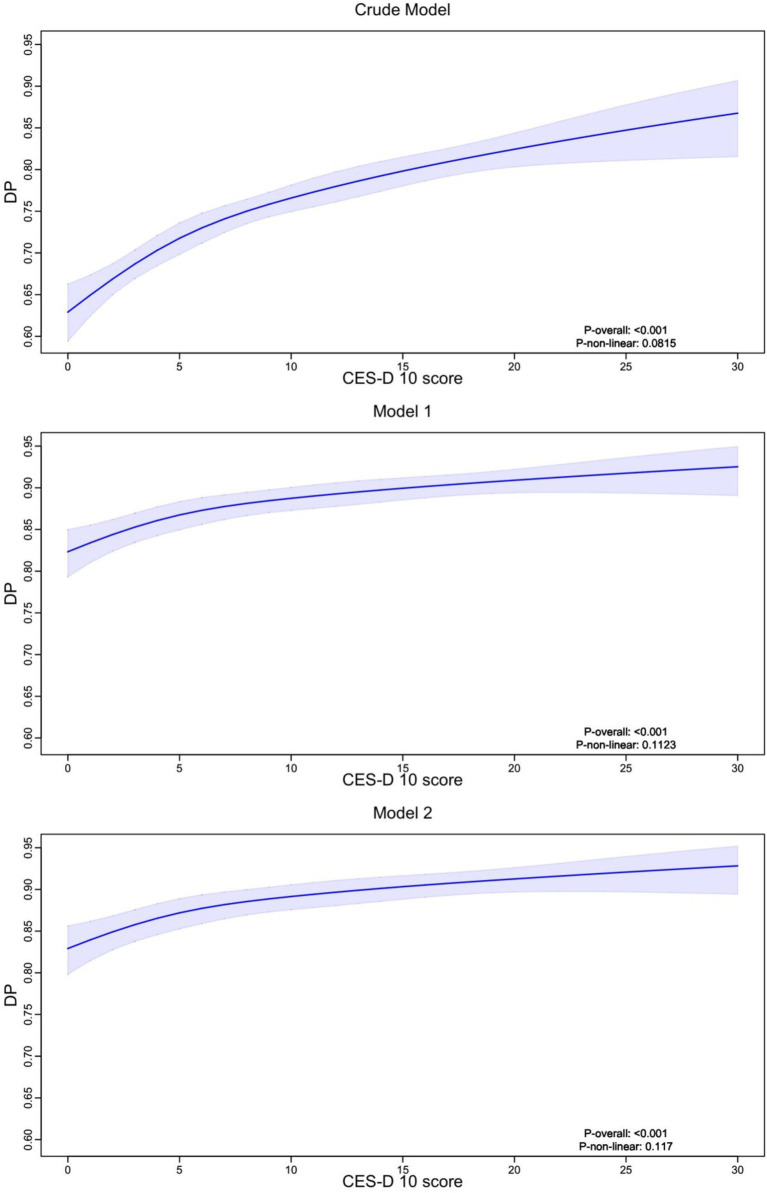
Nonlinear relationship between depression scores and dementia probability. RCS analysis with 4 knots was used to explore the nonlinear relationship between CES-D 10 score and the probability of cognitive impairment or dementia (DP). Three models were presented: a Crude Model (unadjusted), Model 1 adjusted for demographic factors, and Model 2 further adjusted for lifestyle factors and chronic diseases. The results indicate a positive association, where higher depression scores are linked to an increased probability of dementia, with shaded areas representing the 95% confidence intervals. DP, Dementia Probability.

According to the results (Figure 4), depression scores consistently decreased as social activity total scores increased across all models, suggesting a negative association between social activity participation and depressive symptoms. This relationship remained robust across all models (P-overall <0.001).

The relationship exhibited non-linear features in the crude model, with a more pronounced decrease in depression scores at higher social activity levels (>20 points). However, after adjusting for covariates (Models 1 and 2), the relationship became more gradual. As social activity scores increased from 0 to 40, depression scores decreased from approximately 0.95 to 0.85, indicating a modest but significant effect of social activity on depressive symptoms.

The confidence intervals remained relatively narrow for social activity scores between 0–30, but widened at higher scores (>30), suggesting less precise estimates at the upper range of social activity participation. This pattern was consistent across all models, indicating the robustness of the relationship despite adjustment for potential confounders.

### 3.6 Relationship between social activity participation frequency, depression status, and cognitive impairment

#### 3.6.1 Relationship between total social participation frequency scores, depression status, and cognitive impairment

**Figure 5:**
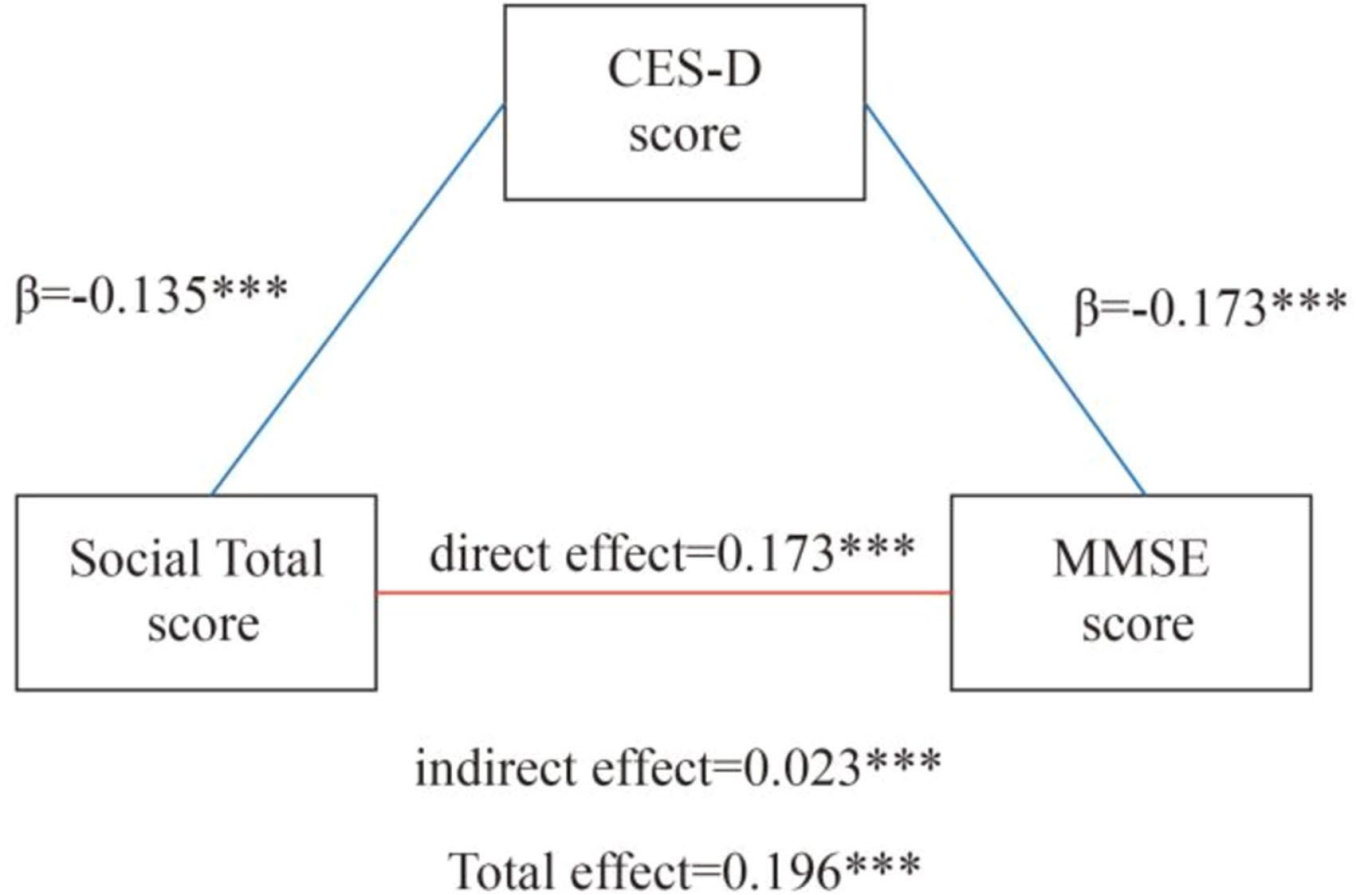
Mediation of depressive symptoms on the pathway between social participation and global cognition. *** indicates *p* < 0.001.

A structural equation model (SEM) analysis of the total social activity participation score revealed several statistically significant results (Figure 5).

The social total score showed a significant negative direct effect on CES-D10 scores (*β* = −0.135, *p* < 0.001), indicating higher social engagement is associated with lower levels of depressive symptoms. A direct positive effect was found between social activity and cognitive impairment (*β* = 0.173, *p* < 0.001).

An indirect effect on cognitive impairment was observed through CES-D10 scores (*β* = 0.023, *p* < 0.001). Higher social interaction scores were associated with lower CES-D10 scores, which in turn had a significant negative impact on cognitive impairment (*β* = −0.173, *p* < 0.001).

The total effect of social interaction on cognitive impairment was significant (*β* = 0.196, *p* < 0.001), emphasizing the comprehensive impact of social engagement on cognitive health.

The model demonstrated excellent fit indices (CFI = 1, TLI = 1, RMSEA = 0, SRMR = 0), supporting the reliability of these findings.

In summary, these results confirm the importance of social engagement for cognitive function, both directly and indirectly through reducing depressive symptoms as measured by CES-D10. These findings provide valuable insights for developing interventions aimed at maintaining cognitive health in older adults through promoting social engagement and addressing mental health.

#### 3.6.2 Impact of different social activity total scores on depression and cognition

**Figure 6:**
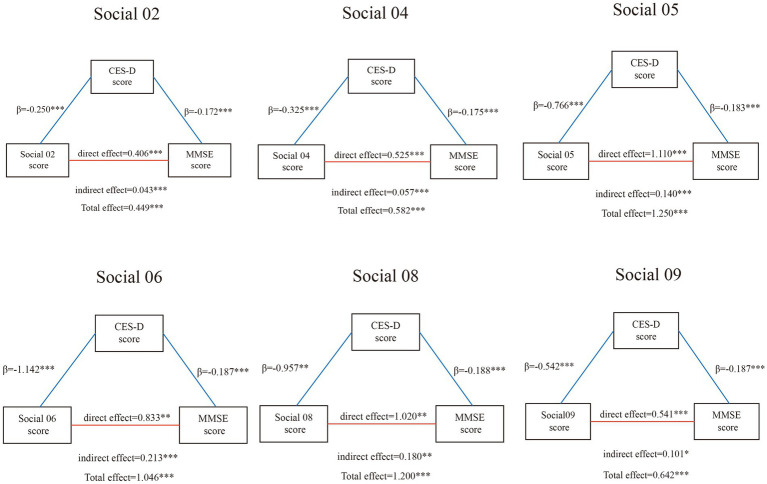
Mediation of depressive symptoms on the pathway between different social activity and global cognition. *** indicates *p* < 0.001, ** indicates *p* < 0.01, * indicates *p* < 0.05.

The SEM analysis of participation frequency in nine social activities revealed several significant patterns (Figure 6). Community-based activities, particularly participation in community organizations (Social 05) and training programs (Social 08), showed the most substantial effects. Community organization participation demonstrated the strongest direct positive impact on cognitive function (MMSE score; *β* = 1.110, *p* < 0.001) and indirectly improved cognitive performance by alleviating depressive symptoms (CES-D 10 score; indirect effect *β* = 0.140, *p* < 0.001).

Other social activities exhibited diverse influences. Volunteer work (Social 06) and social/athletic club participation (Social 04) also demonstrated significant impacts on depression and cognition, with substantial effect sizes (total effects *β* = 1.046 and *β* = 0.582, respectively, both *p* < 0.001). Community club activities and games (Social 02) showed moderate but significant effects (total effect *β* = 0.449, *p* < 0.001), while stock investment activities (Social 09) demonstrated smaller but still significant effects (total effect *β* = 0.642, *p* < 0.001).

Depressive symptoms consistently played a crucial mediating role across most models, linking social activities and cognitive function. This highlights the key bridging role of mental health between social engagement and cognitive function. However, some activities, such as casual conversations (Social 01) and caregiving (Social 07), showed no significant effects on either cognitive function or depression.

Notably, the nature of social activities appeared to influence their effects. Activities involving structured community engagement and learning (like community organization participation and training programs) showed stronger effects compared to casual social interactions or individual assistance activities. This pattern suggests that the complexity and structure of social engagement may be crucial factors in its cognitive benefits.

Most models demonstrated significant direct, indirect, and total effects, with particularly strong evidence for structured community activities. The models showed excellent fit indices (CFI = 1, TLI = 1, RMSEA = 0, SRMR = 0), enhancing confidence in the reliability of the results.

In conclusion, these findings emphasize the importance of structured social engagement, especially activities involving community participation and learning, in maintaining and improving cognitive function and mental health in older adults. The findings further demonstrate the important mediating role of depressive symptoms in the association between social activities and cognitive performance, offering valuable insights for designing interventions that support healthy aging. The results particularly highlight the potential benefits of promoting participation in community organizations, educational programs, and volunteer activities among older adults.

## 4 Discussion

The analysis using the CHARLS database reveals complex relationship patterns among social activities, depression, and cognitive function in older adults. The data demonstrates differential relationships between various types of social activities and cognitive function, mediated by depressive symptoms, which is same with research from Chen (16). Structured community-based activities showed the strongest positive effects on cognitive function, with community organization participation demonstrating the largest total effect (*β* = 1.250, *p* < 0.001), followed by participation in training programs (*β* = 1.200, *p* < 0.001) and volunteer work (*β* = 1.046, *p* < 0.001). These effects operated both directly on cognitive function and indirectly through reduced depressive symptoms (26, 27). However, not all social activities showed equal benefits: casual social interactions and caregiving activities demonstrated no significant effects on cognitive outcomes. This pattern suggests that the impact of social activities on cognitive function varies substantially based on the nature and structure of the activity, with organized, community-based activities showing particularly robust benefits (28).

### 4.1 Activity-specific effects and implications

Analysis reveals distinctive patterns in how different types of social activities influence cognitive function among older adults. Specifically, activities incorporating structured community engagement demonstrated superior protective effects. Community organization participation and training programs showed the strongest associations with cognitive preservation (*β* = 1.110 and *β* = 1.020 respectively), significantly outperforming casual social interactions and individual assistance activities. This finding aligns with previous research showing that organized social networks combining structured activities with cognitive engagement were more effective in preventing cognitive decline (29, 30).

The protective effects varied systematically across demographic characteristics. Education level showed relatively stable protective effects (OR: 0.933–0.967), suggesting that the benefits of social engagement transcend educational background (31, 32). Gender analysis revealed slightly stronger protective effects in males compared to females (OR: 0.960 vs. 0.945) (33, 34). Geographic differences were notable, with rural residents demonstrating stronger protective effects compared to urban dwellers (OR: 0.967 vs. 0.955) (35–37), which is different with Feng’s research (32). This discrepancy might be attributed to differences in the types of social activities available and commonly practiced in rural versus urban settings (34, 38).

Our research findings extend previous studies by demonstrating that the level of structure and organization during social activities may be a crucial determinant of cognitive protection. The results suggest that activities combining social interaction with structured community engagement may optimize cognitive benefits (39). This is particularly evident in the strong effects observed for community organization participation and training programs. This perspective is supported by findings from the Survey of Health, Ageing and Retirement in Europe (40), which similarly demonstrated that structured social activities were associated with better cognitive outcomes among older adults across European countries (41).

### 4.2 Depression as a mediating mechanism

The study found that depression plays a key mediating role in how social activities influence cognitive function. According to our results, the relationship between social activities and depression showed significant non-linear patterns, with depression scores decreasing as social activity participation increased (16). The relationship was more pronounced at higher social activity levels (>20 points) in the crude model, though this became more gradual after adjusting for covariates. As social activity scores increased from 0 to 40, depression scores decreased from approximately 0.95 to 0.85, indicating a modest but significant effect of social activities on depressive symptoms (42).

The relationship between depression and cognitive impairment demonstrated strong non-linear features (P-non-linear <0.001), with a steeper increase in cognitive impairment risk observed at lower depression scores (0–10 points) and a more gradual increase at higher scores (10–30 points). As depression scores increased from 0 to 30, the probability of cognitive impairment increased substantially from approximately 0.60 to 0.90, with this pattern remaining consistent across all adjusted models.

This complex mediating relationship is particularly evident in the structural equation modeling results, where different types of social activities showed varying degrees of indirect effects through depression. Volunteer work demonstrated the strongest indirect effect through depression (*β* = 0.213, *p* < 0.001), followed by training programs (*β* = 0.180, *p* < 0.01) and community organization participation (*β* = 0.140, *p* < 0.001). This suggests that social activities help reduce depressive symptoms by enhancing social connections and offering emotional support (43), which aligns with previous research findings in Canada (44). The significant mediating effects across multiple types of structured social activities underscore the crucial role of mental health in the pathway between social engagement and cognitive function.

### 4.3 Effects of different social activity types and depression mediation on cognitive function

This study analyzed the relationship between participation frequency in nine different types of social activities and cognitive function. Structural equation modeling showed that structured community activities, particularly community organization participation (*β* = 1.110) and training programs (*β* = 1.020), had the most significant positive correlations with cognitive function and indirectly influenced cognition by reducing depression levels (indirect effects *β* = 0.140 and *β* = 0.180 respectively). These findings align with a longitudinal study conducted in Chicago (45). Volunteer work also demonstrated substantial effects (direct effect *β* = 0.833, indirect effect *β* = 0.213) (46–48). In contrast, casual social interactions and caregiving activities showed no significant effects on either cognitive function or depression. These findings support the importance of structured social engagement, suggesting that activities involving organized community participation can more effectively maintain and promote cognitive function in older adults.

Depression consistently played a crucial mediating role in the relationship between most social activity frequencies and cognitive function. The mediating effects were particularly strong for volunteer work (*β* = 0.213) and training programs (*β* = 0.180), highlighting the importance of assessing and managing mental health when formulating cognitive function protection plans for community-dwelling older adults (16, 37). The study provides empirical evidence for developing targeted interventions that combine emotional well-being promotion with structured social engagement to comprehensively maintain cognitive function in the older adult population.

### 4.4 Practical and theoretical implications for cognitive health interventions

Our findings have significant implications for both public health practice and theoretical research. From a practical perspective, the study highlights the role of structured social activities as crucial intervention measures for preventing cognitive decline, aligning with recent Chinese policy initiatives. The State Council’s “Opinions on Strengthening Aging Work in the New Era” emphasizes promoting high-quality social participation among older adults (49), while “The 14th Five-Year Plan” outlines strategies for active aging (50). Our results, particularly regarding the benefits of cognitive-stimulating activities, directly support these policy directions and suggest that community programs should prioritize activities combining social interaction with cognitive engagement. Additionally, the study recommends integrating mental health management into cognitive function protection plans to establish comprehensive intervention models, as demonstrated by the significant mediating effects of depression across multiple activity types.

From a theoretical perspective, the study identifies consistent relationships between social activities and cognitive function and reveals the crucial mediating role of depression in cognitive protection. The findings enhance our understanding of how social engagement influences cognitive health through both direct and indirect pathways, with structured activities showing particularly robust effects. These findings provide theoretical and practical support for fostering healthy aging in an increasingly aging society.

### 4.5 Research methodology evaluation

#### 4.5.1 Study strengths

This investigation demonstrates several significant methodological strengths that enhance its scientific contribution. The analysis utilizes data from 6,802 CHARLS participants, providing substantial statistical power and broad demographic representation across China’s older adult population. The application of structural equation modeling effectively captured complex relationships between variables, revealing both direct effects and mediating pathways across different types of social activities. The excellent model fit indices (CFI = 1, TLI = 1, RMSEA = 0, SRMR = 0) across all analyses strengthen confidence in the reliability of the findings.

#### 4.5.2 Study limitations

While this study has significant methodological strengths, several limitations should be considered. The sensitivity analysis revealed important selection bias concerns, as the included participants demonstrated significantly higher participation rates across most social activity types compared to excluded individuals. The included group was slightly younger (57.71 ± 8.39 vs. 57.93 ± 9.16 years) and had a higher proportion of females (55.27% vs. 44.73%). These disparities suggest our findings may be more applicable to slightly younger and more female older adult populations who are more socially active.

The “healthy participant effect” (51, 52) might lead to an overestimation of the protective effect of social activities on cognitive function. Additionally, while the structural equation modeling provided robust analysis of relationships, the cross-sectional nature of some analyses limits causal inference. Future research should prioritize longitudinal designs and strategies to include less socially active older adult populations to ensure more generalizable findings.

## 5 Conclusion

Through analysis of CHARLS data, this study provides robust evidence for the complex relationships between social activities, depression, and cognitive function in older Chinese adults. Structural equation modeling demonstrated that social engagement impacts cognitive health through both direct and indirect pathways, with depression serving as a crucial mediating factor. The relationship between social activities and cognitive function showed consistent protective effects across various demographic subgroups.

Our findings reveal that structured community-based activities, particularly community organization participation and training programs, offer superior protective effects against cognitive decline compared to casual social interactions. Volunteer work also demonstrated substantial benefits. The differential effects across demographic groups, including slightly stronger protective effects in rural areas and among male participants, provide valuable insights for tailoring interventions to specific populations.

The mediating role of depression was consistently demonstrated across structured social activities, with the strongest indirect effects observed in volunteer work and training programs. This highlights the interconnected nature of mental health and cognitive function in aging populations. As social activity participation increased, depression scores showed significant decreases, while higher depression scores were associated with increased cognitive impairment risk in a non-linear pattern.

These results have significant implications for public health policy and clinical practice in China’s rapidly aging society, supporting the development of community-based programs that emphasize structured social engagement while addressing mental health concerns. The findings contribute to understanding modifiable factors that may help preserve cognitive function in later life, particularly highlighting the importance of organized community participation and educational programs. This provides a scientific foundation for developing targeted interventions to promote healthy aging globally, with special attention to the role of structured social activities and mental health management.

## Data Availability

The datasets presented in this study can be found in online repositories. The names of the repository/repositories and accession number(s) can be found at: the CHARLS database provided the data for this investigation. Visit http://opendata.pku.edu.cn/dataverse/CHARLS to get CHARLS data.
